# Vertically Grown Bioinspired Diphenylalanine Nanowire-Coated Fabric for Oil–Water Separation

**DOI:** 10.1021/acsaenm.4c00381

**Published:** 2024-08-05

**Authors:** Noah Hann-Deschaine, Neha M. Viradia, Jeiko J. Pujols, Sarah Miller, Ramesh Y. Adhikari

**Affiliations:** † Department of Physics & Astronomy, Colgate University, 13 Oak Drive, Hamilton, New York 13346, United States

**Keywords:** diphenylalanine, parahydrophobic, peptide self-assembly, bioinspired nanowires, oil−water separation, fabric mesh

## Abstract

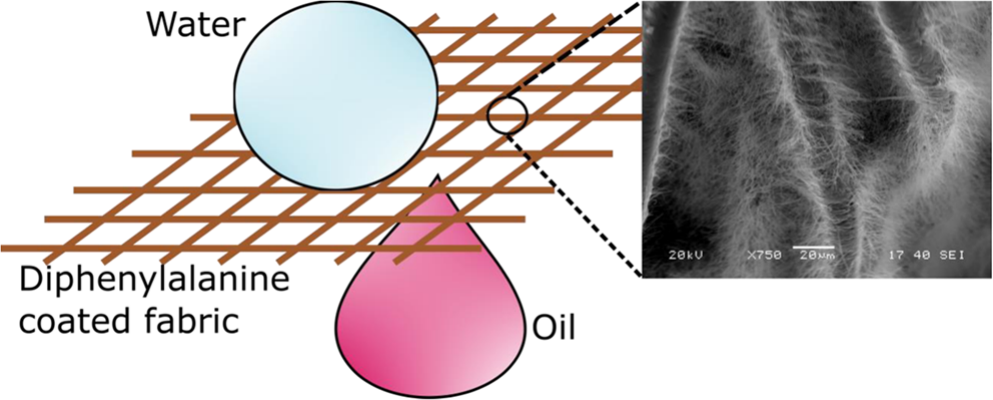

Due to the pervasive use of oil for energy and other industrial applications, solutions to oil–water separation have received a great deal of attention lately to address the environmental damage of oil spills and groundwater contamination. However, many of these separation methods are materially expensive and environmentally hazardous, require elaborate fabrication, or rely on large amounts of energy to function. Herein, we provide an effective low-cost method for oil–water separation based on the hydrophobicity induced by self-assembled bioinspired diphenylalanine peptide nanowires grown on polyester fabric. This modified polyester fabric mesh exhibits parahydrophobicity and oleophilicity due to the hierarchical nano-to-microscale surface roughness. This mesh also achieves consistent high water separation efficiencies of over 99% and an ultrahigh oil flux of up to 26.7 ± 5 kLm^–2^·h^–1^. The growth of bioinspired peptide-based nanostructures on fabrics using facile technique and their application in oil–water separation presents the potential for using bioinspired materials for environmental remediation while minimizing environmental footprint.

## Introduction

Oil is an essential part of the energy source in the world, accounting for more than 30% of primary global energy sources.^1^ It is the primary source of energy for internal combustion engines in automobiles, jet engines, industrial processes, and heating. Oil and its derivates are also used for other purposes such as lubricating machinery, which all find their way into wastewater systems. In addition, every now and then, massive amounts of oil are spilled into water sources due to accidents during the operation of offshore rigs, pipelines, or oil tankers. For example, in one of the most recent major oil spills in the United States, more than a million gallons were spilled into the Gulf of Mexico off the Louisiana coast in November 2023 due to damage in a pipeline.^2^ At other times, even larger-scale spills such as the 2010 Deepwater Horizon spill have resulted in the release of millions of barrels of oil into the water.^3^ The release of these hydrocarbons into the water systems results in ecological damage in both freshwater and marine environments and brings economic hardships to the communities depending on those water sources.^4^ Among several techniques developed to address this issue, research and development of various hydrophobic surfaces and membranes have drawn growing attention.^5^

Hydrophobic materials are characterized by nanoscale surface roughness that results in poor wettability, contact angles larger than 90°, extremely efficient water repellency, and typically poor adhesiveness to water.^5−7^ Various physical and chemical methods have been used to develop hydrophobic and oleophilic surfaces.^8−11^ Physical methods involve techniques such as plasma treatment,^10,12^ template imprinting,^13,14^ spin-coating,^15^ spraying,^16^ and electrospinning.^17^ Chemical methods involve techniques such as self-assembly,^18^ electrochemical methods,^19^ sol–gel methods,^20^ and solvothermal methods.^21^ Most of these techniques require a significant amount of energy, are difficult to scale up, or are constructed with materials that are unable to be reused, recycled, or discarded in environmentally friendly manners.

In order to address some or all of these challenges, there have been various studies in which fabrics have been used to construct oil–water separating membranes. Fabrics are inexpensive and produced in large volumes, and over 60% of them are synthetic.^22^ Increased consumption of textiles and shorter garment longevity have resulted in millions of tons of fabric-based waste every year,^23^ most of which are either discarded in landfills or burned for energy.^24^Therefore, reusing these discarded fabrics would not only provide a cost-effective alternative but also help offset some of the environmental impacts. Hydrophobic fabric-based meshes^25−27^ have emerged as a viable option for oil–water separation due to their flexibility, separation efficiency, and ease of manufacturing. Hydrophobicity in these fabrics is introduced by various methods to induce surface modifications such as by chemical functionalization of the fabric surfaces,^28^ plasma treatment,^29^ dip coating,^27,30,31^ etc., with the growing number of publications focusing on identifying methods that involve simple fabrication methods and are cost-effective. However, hydrophobicity in most of these fabrics is achieved by coating various chemicals on the fabric surfaces that are not necessarily environmentally friendly and/or nontoxic.

While there have been various reports of use of bioinspired methods and biobased mesh-like substrates to create hydrophobic surfaces for oil–water separation, use of entirely bioinspired and biobased nanostructures to make mesh substrate surface hydrophobic for oil–water separation are limited.^32,33^ For example, Li et al. developed a hydrophobic cotton fabric-based oil–water separator by covalently bonding polydopamine, a biomimetic molecule, coated with silicon dioxide nanoparticles that are not biomimetic.^34^ Li et al. developed an oil–water separation mesh by applying bioderived potato residue powder on a stainless steel mesh with the help of waterborne polyurethane, a non-bioderived binder.^35^ Zhang et al. developed an oil–water separator by coating rough copper mesh with chitosan, a bioinspired molecule derived from chitin, by mixing with poly(vinyl alcohol), which is not biobased.^36^ Here, we report the development of hydrophobic fabric mesh for oil–water separation by vertically growing nanowires entirely composed of bioinspired peptide diphenylalanine (FF) on fabric threads without the need for non-biobased material to act as a binder or part of the composite in the final product.

FF is one of the most well-studied short peptides found as the core recognition motif of Alzheimer’s β-amyloid polypeptides.^37,38^ Due to its aromaticity and charges,^39,40^ FF can be self-assembled into rigid nanowires^41−43^ by using solution-based^44−46^ or vapor-based methods.^47−49^ Using the alanine vapor-based deposition method, it was previously reported that the vertical growth of FF nanowires can result in hydrophobic surfaces.^47,50^ In this paper, we report that the alanine vapor-based method can also be used to grow vertical FF nanowires on a fabric, which results in the formation of parahydrophobic and oleophilic fabric mesh. We also report that such a hydrophobic fabric can be used as a highly efficient (>99%) oil–water separating mesh with a long shelf-life without a significant degradation in the oil separation efficiency (>97%) even after a year. These meshes also allow for an ultrahigh oil flux rate of up to 26.7 ± 5 kLm^–2^·h^–1^, which is one of the highest values that has been reported for oil–water separation membranes based on hydrophobic meshes.

## Materials and Methods

### Materials

Diphenylalanine (FF) anhydrous powder (CAS-No: 2577-40-4), 1,1,1,3,3,3-hexafluoroisopropanol (HFIP) > 99% (CAS-No: RN 920-66-1), and aniline (CAS-No: 62-53-3) were purchased from Fisher Scientific, USA. Dye for oil, Sudan IV (CAS-No: 85-83-6), was bought from Sigma-Aldrich, USA. Polyester-based faux burlap fabric was purchased from Amazon (13 × 108 Khaki, LuoluoHouse Burlap). Olive oil was purchased from a local grocery store in Hamilton, New York, USA. Petrol and diesel were purchased from a local gas station in Hamilton, New York, USA.

### Contact Angle Measurements

Sessile contact angle measurements were taken on an Ossila Contact Angle Goniometer (L2004A1) with the help of software provided by the manufacturer. For the contact angle measurements, we used 10 μL of water dropped onto the surfaces with the help of a syringe.

### Surface Analysis

We carried out attenuated total reflection ATR-FTIR spectroscopy by using a Nicolet 6700 FTIR spectrometer. The scan resolution was 2 cm^–1^ with 64 scans within a scan range of 650–4000 cm^–1^. X-ray diffraction (XRD) measurements were carried out on a Philips PW3040 X-ray diffractometer with X’Pert software for extracting the data. The instrument uses Cu Kα radiation with a wavelength of 1.54 Å. Each 1 cm × 1 cm piece of the pristine fabric and FF-coated fabric was placed on a glass slide with the help of double-sided tape before placing the glass slides in the diffractometer for measurements.

### Separation Efficiency and Flux Measurements

Separation efficiency measurements were taken by recording the mass of oil, water, and FF-coated faux-burlap-based mesh before and after the separation was carried out. Oil separation efficiency (*Q*_se_) was carried out by using , where *m*_f_ is the mass of oil that was reclaimed after the separation and *m*_i_ is the initial mass of the oil used to prepare the oil–water mixture. Oil flux (F) was calculated by using *F* = *V*/*At*, where *V* is the volume of oil collected through the fabric mesh boat through its surface area *A* over the duration of time *t*. All oil–water separation measurements were carried out by first coloring the oil with Sudan IV and then mixing 10 mL of oil with 10 mL of water.

## Results and Discussion

### Vertical Growth of FF Nanowires on the Si Substrate

**Figure 1:**
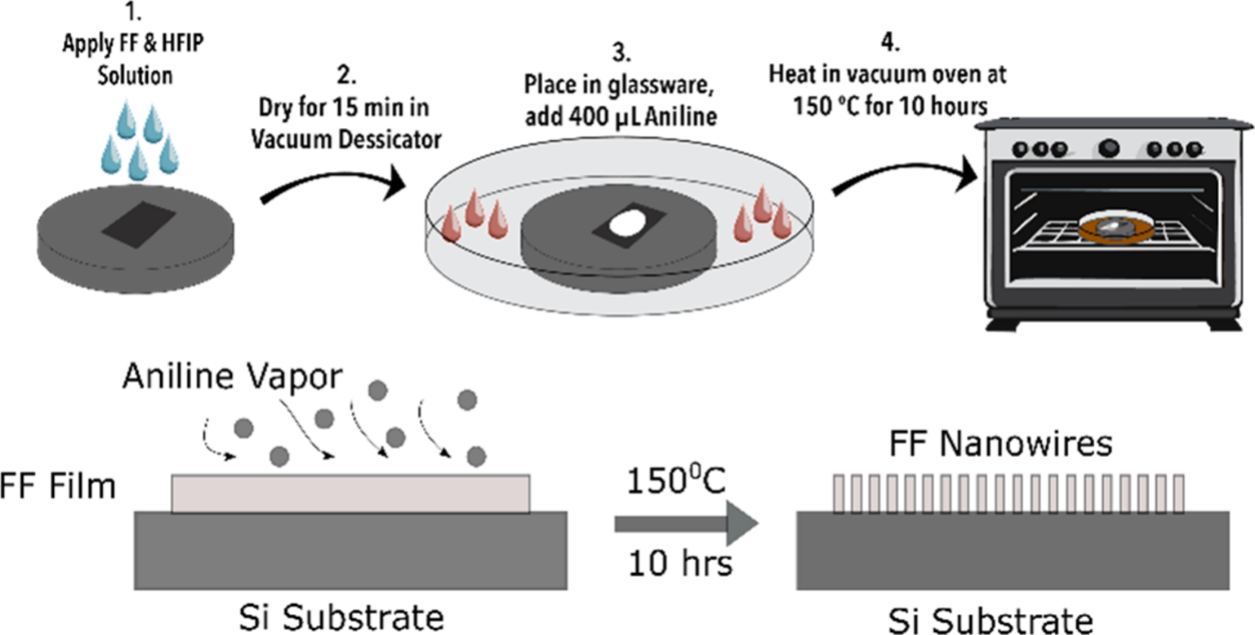
Fabrication process of vertically grown FF on a silicon substrate.

**Figure 2:**
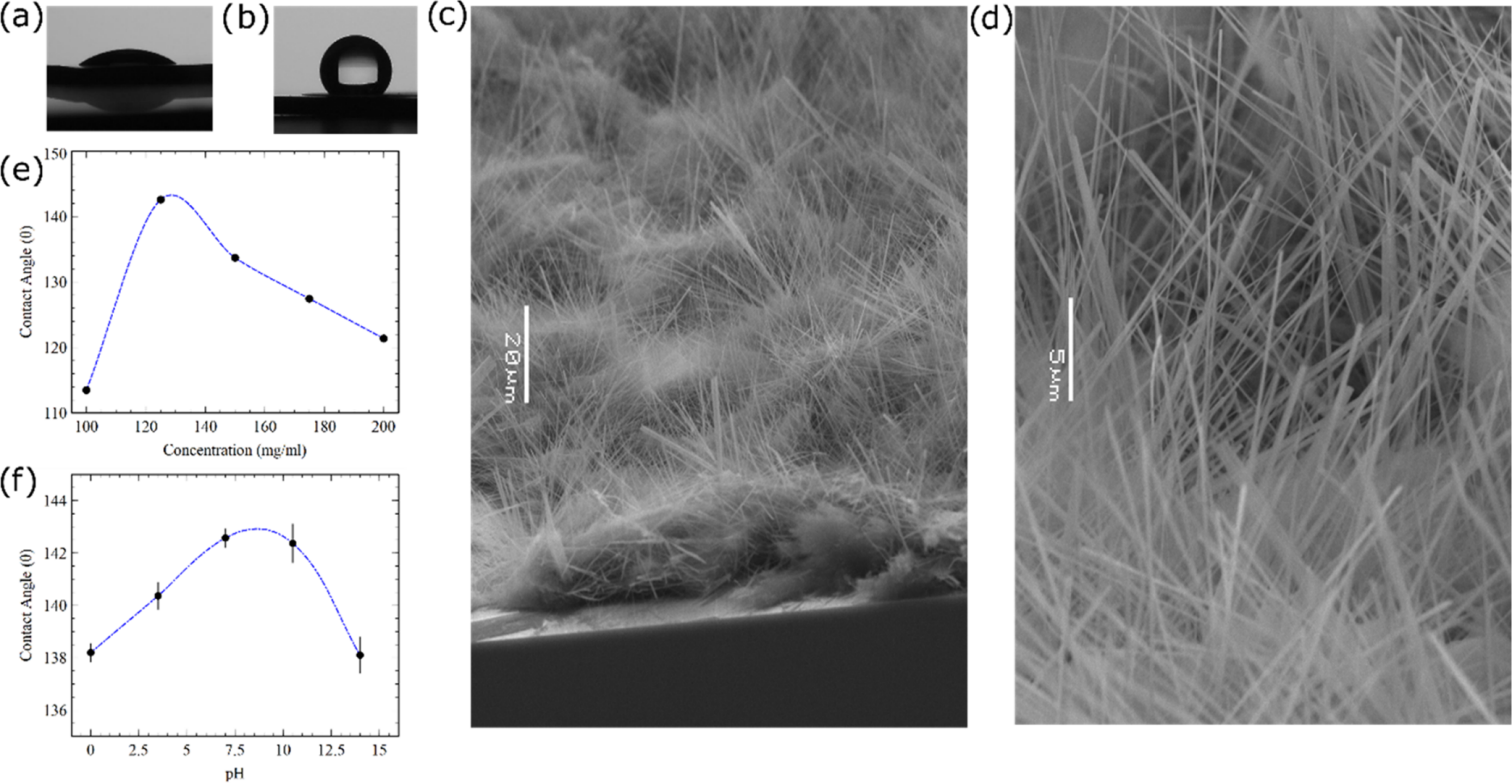
Water drop on silicon dioxide substrate with a top oxide layer (a) before nanowire growth and (b) after nanowire growth. (c) SEM image of the nanowire growth on the silicon substrate (scale bar: 20 um). (d) Magnified image of the area with nanowire growth (scale bar: 5 um). The contact angle of water droplets on the hydrophobic surface prepared on a Si substrate (d) with increasing FF concentration during preparation and (e) as a function of pH of the water droplet on the hydrophobic surface prepared from 125 mg·mL^–1^ solution. The blue lines are guides for the eye.

We used a modified version of the fabrication method (Figure 1) of the vertical growth of FF nanowires reported elsewhere.^47,50^ We prepared a solution containing FF on HFIP and drop cast it on a clean silicon wafer substrate. We then placed the substrate in a vacuum desiccator for 15 min. Then, we removed the substrate from the desiccator and placed it on an elevated platform in a glass Petri dish with 420 μL of aniline. We then covered the dish with its cap and sealed it with aluminum foil before placing it in a vacuum oven at 150 °C for 10 h. Previous studies have suggested that drop casting the FF-HFIP solution on a substrate creates a thin amorphous film of FF, which undergoes nucleation and then self-assembly as the sample is aged at higher temperatures in the aniline vapor environment, which acts as a hydrogen bond donor.^47,50^ The area with FF nanowire growth appeared white (Figure S1) and was significantly more hydrophobic than the silicon substrate with no FF nanowire growth (Figure 2a,b). The SEM image of the growth area demonstrated the presence of vertical nanowire growth (Figure 2c). Upon closer inspection, it was clear that the nanowires were tens of micrometers tall, with the diameter in the range of tens of nanometers, suggesting that these vertically grown nanowires have a high aspect ratio (Figure 2d).

We also investigated whether varying the concentration of FF in the HFIP solution has any effect on the nanowire growth and hydrophobicity. We measured the resulting contact angle on these surfaces and observed that the contact angle increases initially as the concentration of FF is increased from 100 to 125 mg·mL^–1^, reaching 142.57 ± 0.36° (Figure 2e). This value is slightly lower than the 150° benchmark^51,52^ widely used for superhydrophobicity but higher than the previously reported contact angle for the similar procedure of the aniline-based FF growth.^47^ However, after a further increase in the concentration of the FF, the contact angle decreased linearly. Among other parameters essential for creating hydrophobic surfaces, the roughness of the surface is an important one. Commonly used models to quantify hydrophobicity such as Wenzel’s model suggest that increased roughness increases the contact angle due to an increase in the solid–liquid interfacial surface tension.^6^ With the initial increase in the concentration of FF, there is an increase in the roughness of the surface with the increasing density of vertically grown FF nanowires. Upon further increase in the concentration of FF, the nanowires become more and more closely packed together, lowering the roughness factor of the surface and, therefore, lowering the contact angle.

We further recorded the contact angle by varying the pH of the water droplet (Figure 2f). The largest contact angle values were observed for the water at neutral pH, and the contact angle diminished for the water droplet with either a higher or lower pH. However, the change in the contact angle was within 3% of the maximum contact angle at a neutral pH, suggesting that the hydrophobic surfaces based on FF can withstand extreme pH conditions. Furthermore, while the vertical growth of FF nanowires makes surfaces hydrophobic, we observed that these surfaces also become oleophilic (Figure S2) as the contact angle of an oil drop diminishes exponentially over time as it spreads out when it lands on the hydrophobic surface (Figure S3).

### Vertical Growth of FF Nanowires on a Faux Burlap Fabric

**Figure 3:**
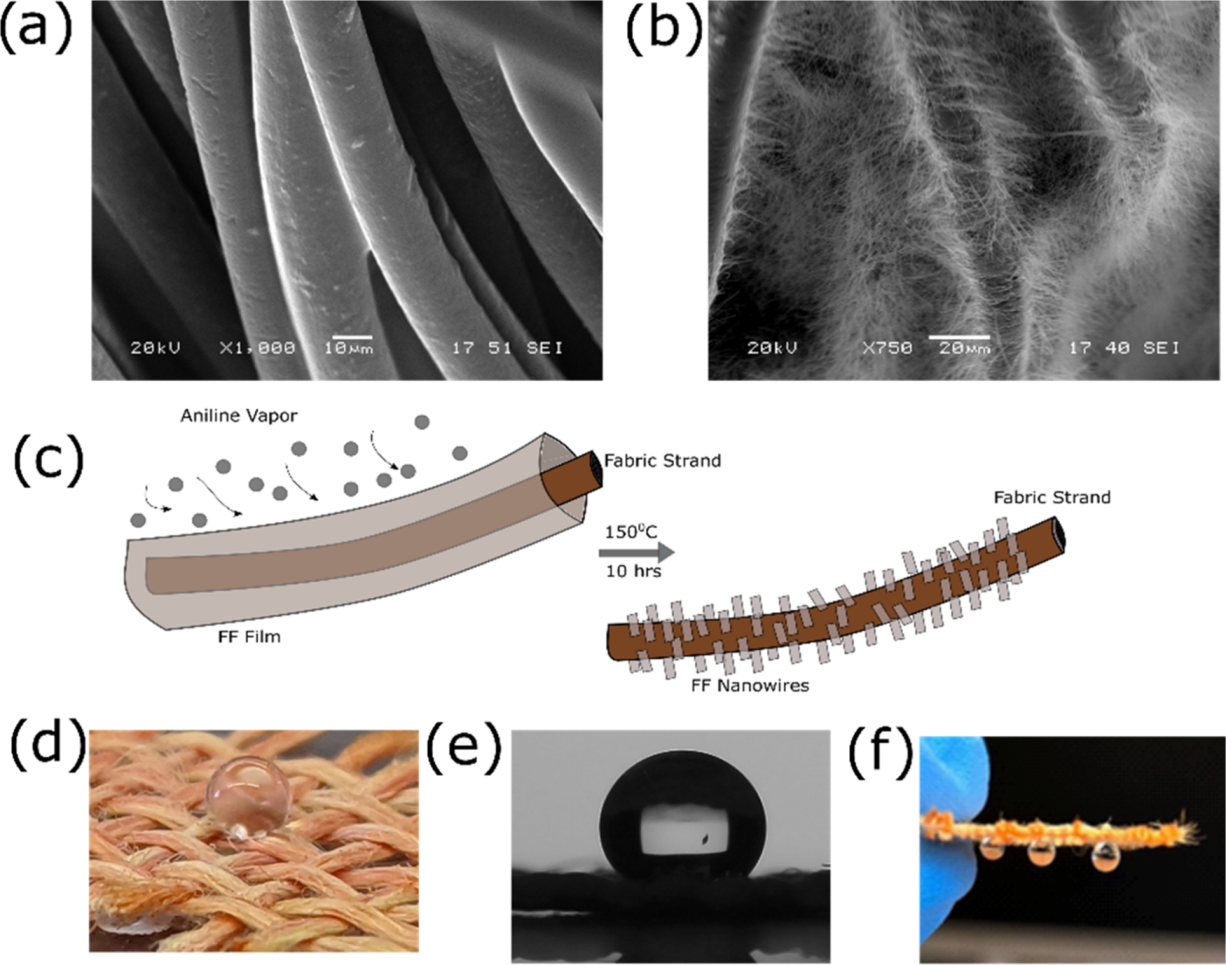
SEM images of (a) pristine fabric strands (scale bar: 10 um) and (b) fabric strands with FF nanowires grown on the surface (scale bar: 20 um). (c) Sketch of the vertical nanowire growth process on a fabric strand. (d) Optical image of a water droplet on the fabric with FF nanowire coating, and (e) side view of the water droplet on the fabric with FF nanowires as observed from a goniometer, which was used to calculate the contact angles. (f) Demonstration of the parahydrophobicity on the FF nanowire-coated fabric as water droplets hung onto the hydrophobic fabric surface even after the fabric was turned over.

After optimizing the parameters for FF nanowire growth that results in hydrophobic and oleophilic surfaces, we investigated whether FF nanowires can be grown on fabric strands as well to achieve the same surface properties. We immersed a piece of polyester faux burlap fabric into a 125 mg·mL^–1^ FF-HFIP solution and then repeated the process used for growing the nanowires on silicon substrates (Figure 1). Upon inspection of the pristine fabric under SEM, the fabric threads appeared smooth, as expected (Figures 3a and S4a,b). However, the threads of fabric that had undergone the nanowire growth process had a dense set of nanowires on the surface (Figures 3b and S4c,d), demonstrating the successful growth of FF nanowires on the fabric. Just as in the case of the vertical growth of FF nanowires on the Si substrate, exposure of the FF-HFIP solution-coated fabric to aniline vapor at 150 °C resulted in the vertical growth of nanowires on the fabric strand surfaces, indicating that the same self-assembly mechanism is in play in this situation as well (Figure 3c). Despite the presence of significant gaps between fabric strand bundles (Figure S5) through which the water would normally seep, the presence of nanowire coatings on the fabric strands had now made the fabric hydrophobic (Figure 3d,e). We measured that the contact angle between the water droplet and the fabric was 132.92 ± 0.39°. In addition, as in the case of the FF grown on a Si substrate, the fabric coated with FF also demonstrated oleophilic behavior as the oil droplet spread throughout the fabric surface as it landed and then made its way through the fabric (Figure S6 and Movie S1). While this process took a few seconds to occur on the nanowire-coated Si substrate, it only took a fraction of a second for the oil to dissipate and pass through the nanowire-coated fabric.

The hydrophobic surfaces that are commonly developed are usually reported to be self-cleaning, as the water drops run off the surface when the hydrophobic surfaces are inclined. Such an effect is commonly called the lotus leaf effect.^6,51^ However, we observed that water drops on the surface of the hydrophobic fabric that we developed do not run off its surface. In fact, the adhesion of the water drop to the hydrophobic surface was so strong that the water drops stayed attached to the surface even after the surface was overturned (Figure 3f). Such behavior with a high level of hydrophobicity in conjunction with a strong pinning/adhesion to the surface is called the rose petal effect or parahydrophobicity.^53,54^ This phenomenon is commonly attributed to the presence of hierarchical micro and nanoscale structures on the surface^53,55^ which applies to the case of the fabric coated with FF nanowires as well since the threads of the fabric represent microscale structures, while the nanowires grown on those threads represent the nanoscale structures.

**Figure 4:**
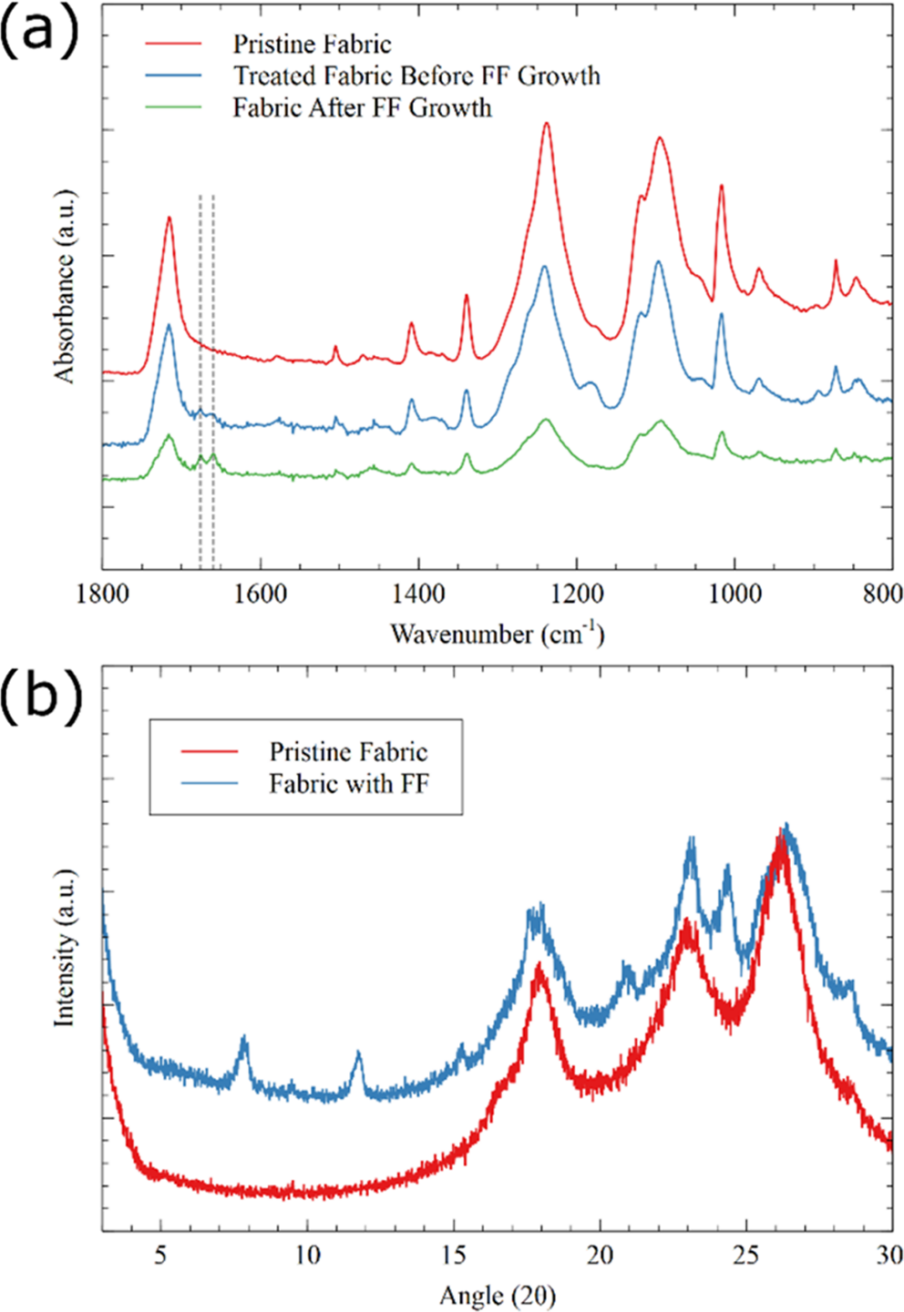
(a) FTIR and (b) XRD spectra of the pristine fabric and the FF-grown fabric. The peaks associated with antiparallel β-sheet (1676 cm^–1^) and α-helical structure (1658 cm^–1^) in vapor-grown FF nanowires in the FTIR spectrum have been indicated by the vertical lines.

We also carried out surface FTIR on the fabric and noticed that the growth of FF nanowires was indicated by well-defined peaks at 1676 and 1658 cm^–1^ (Figure 4a). The former peak is attributed to the antiparallel β-sheet, while the latter peak is attributed to the α-helical structure.^56^ The fabric treated with the FF-HFIP solution but not gone through the vapor-based growth process also demonstrated these peaks, albeit not so well-defined, due to a lack of completely self-assembled FF structures. The pristine fabric demonstrated no peak at that region, suggesting a lack of FF. We also carried out XRD measurements of the pristine fabric and fabric with FF nanowires (Figure 4b). Compared to the peaks for pristine fabric, we observed additional peaks at angles (2θ) of 7.8, 11.7, 15.3, 20.8, and 24.4° for a fabric with FF growth with the corresponding *d* spacings of 11.3, 7.6, 5.8, 4.2, and 3.7 Å, respectively. From these values, it appears that 15.3 and 24.4° are the second- and third-order reflections of 7.8° associated with the *d*-spacing of 11.3 Å, respectively. This *d*-spacing has been reported previously for vapor-grown vertical FF nanowires fabricated using a similar procedure^47^ and is slightly smaller than the *d*-spacing of 13.6 Å reported for FF nanowires fabricated using a solution-based process^38^ and assigned to the symmetric hexagonal ring formation during the self-assembly process.

### FF-Coated Faux Burlap as an Oil–Water Separator

**Figure 5:**
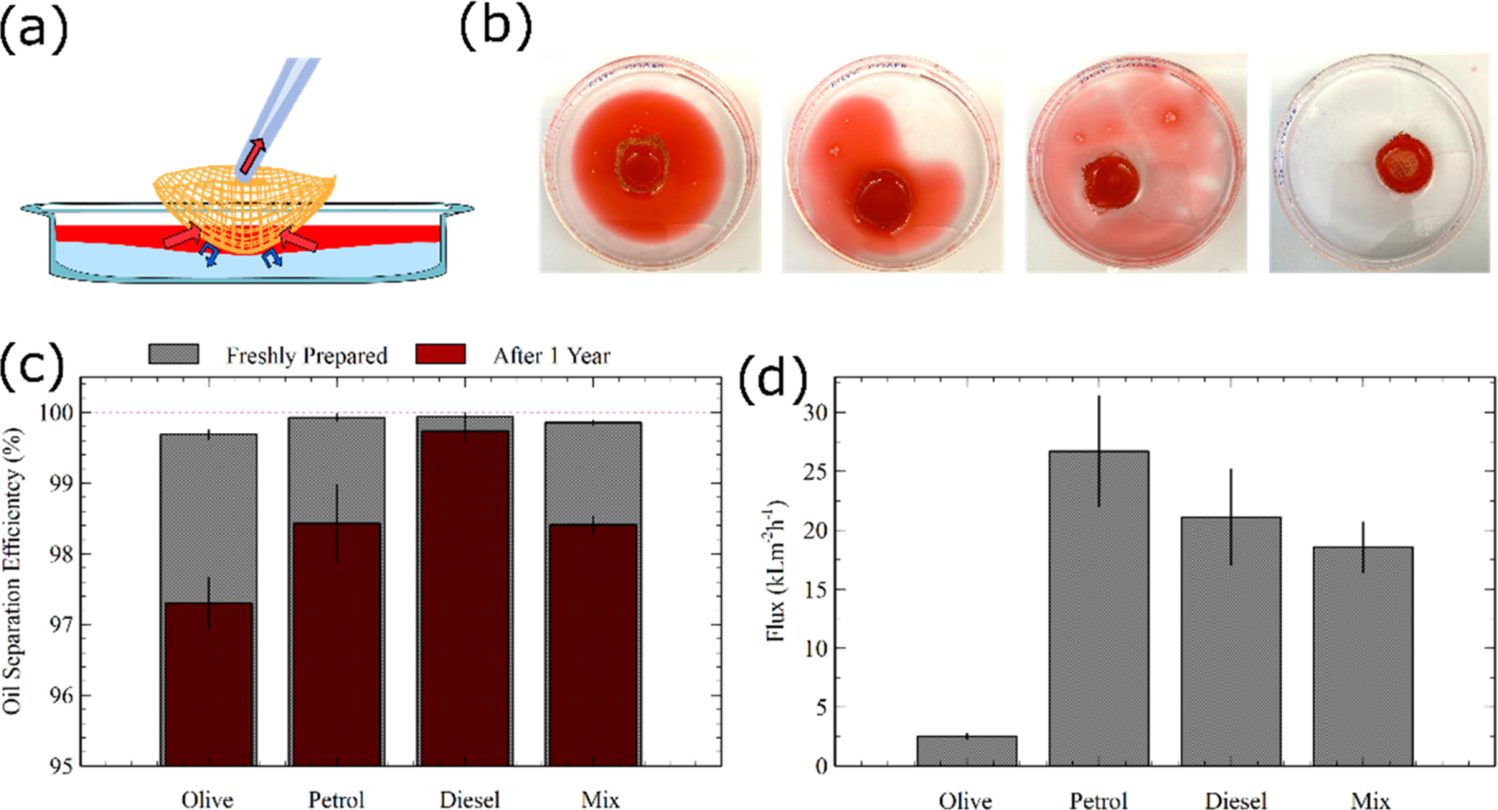
Oil–water separation. (a) Sketch demonstrating the flow of water and oil when the FF-coated boat is immersed in the water. Oil collected in the boat is siphoned away with the help of a syringe. (b) Top view of the olive oil–water mixture as oil is collected from the mixture. The oil is in red. The area with no colors represents water. (c) Separation efficiency of various kinds of oil from water. A freshly prepared nanowire-coated fabric mesh boat is highly efficient, while the fabric mesh boat prepared a year ago still maintains a high degree of efficiency. (d) Flow rate of various kinds of oil into the fabric mesh boat.

With the development of a nanowire-coated hydrophobic and oleophilic fabric, we investigated the possible application of such a fabric as an oil–water separating membrane. For this purpose, we prepared a fabric-based “boat”, i.e., a bowl-shaped mesh (Figure S7). They were prepared by placing the fabric sheets onto a hemispherical aluminum foil mold before applying an FF-HFIP solution. Since the FF growth on the fabric makes it hydrophobic and oleophilic, this shape of the mesh allowed us to dip the hydrophobic boat and move it around in the oil–water mixture to allow the oil to be collected in the boat while keeping the water out (Figure 5a). During this process, oil moves into the boat due to the pressure imbalance, while the water stays outside the boat due to the FF-based hydrophobic coating. The oil can then be reclaimed by simply extracting the collected oil inside the boat with the help of either a syringe or a pump while the boat is still submerged in the water (Movie S2).

To quantify the water separation efficiency using these fabric boats, we colored the oil with Sudan IV so that we could visually differentiate oil from water as the oil is colored in red. We then used the FF-coated boat made from fabric and placed it onto the mixture (Figure 5b). As expected, the oil started getting collected into the boat as the boat was moved around the water surface and the area covered by the oil on the water diminished over time. We carried out the oil–water separation measurements with olive oil, petrol, diesel, and an oil mixture with a 1:1:1 ratio of the three oils used. For all these scenarios, the efficiency with which the original amount of water was retrieved was quite high, with more than 99% of oil retrieved from all of the measurements (Figure 5c). Among these, the lowest efficiency was with olive oil, with 99.69% oil separation efficiency, and the highest efficiency was for diesel, with 99.94% efficiency. After the fabric boat had collected oil from the oil–water mixture, we observed that the oil could stay retained within the boat for over 5 days while the boat was left floating in the water (Figure S8), suggesting that the nanowire coating on the fabric is robust and can effectively maintain oil–water separation for a long duration while the boat is left on the water. We also carried out oil–water separation measurements with FF nanowire-coated mesh boats that were prepared more than a year ago. We observed that the separation efficiency from the old boats was still high, with over 97% separation efficiency for all oil types (Figure 5c). Furthermore, we also observed an ultrahigh level of oil flux values (Figure 5d) through these FF nanowire-coated fabric mesh boats. The lowest oil flux value was at 2.5 ± 0.2 kLm^–2^·h^–1^ for olive oil, while the highest value was at 26.7 ± 5 kLm^–2^·h^–1^ for petrol. We attribute this difference to the higher viscosity of olive oil compared to those of petrol and diesel. Given that the nanowire-coated fabric allowed for the oil drops to permeate through them extremely fast, as mentioned above (Figure S6), these oil flux values appear to be reasonable. It is to be noted that these are toward the higher end of the values reported for using mesh-based oil–water separation technique^30,57−60^ irrespective of the physical or chemical fabrication method or the use of inorganic or bioinspired and biobased materials.

Due to their biocompatibility, the ability to readily self-assemble into nanowires or nanotubes in a solution^44^ or vapor environment,^47^ having robust mechanical properties,^42,43^ and being piezoelectric due to inherently charged sites,^46^ these nanostructures of FF have found their application as substrates for cell cultures,^49^ material to increase surface roughness for supercapacitors,^61,62^ template for conducting polymer nanotubes,^63^ and active material for piezoelectric energy harvesters.^45,46^ Our work reported herein demonstrates yet another application of these FF nanowires as coatings for constructing oil–water separation membranes. The combination of high separation efficiency, extremely high oil flux, and long shelf-life demonstrates that bio-inspired FF nanowire-coated fabric-based hydrophobic mesh can be a robust, reliable, and low-cost alternative to commonly used methods for creating hydrophobic and oleophilic membranes for oil–water separation. The ease of processability of FF and the robust mechanical properties of its self-assembled nanostructures allow for these attributes in FF-coated oil–water separation meshes. Therefore, due to molecular diversity and ease of processing, bioinspired peptide-based materials hold potential for a wide range of applications including their use as coatings for oil–water separation membranes as greener alternative to other organic and inorganic materials.

## Conclusions

We have demonstrated that it is possible to grow bioinspired peptide-based vertical FF nanowires on fabric surfaces with their potential for application as oil–water separation membranes. These FF nanowire-coated fabrics were parahydrophobic. We observed the highest contact angle of 142.57 ± 0.36° with the FF grown on a silicon substrate and 132.92 ± 0.39° with the FF grown on a fabric. The contact angles did not significantly change with the pH of the water, suggesting that these FF nanowire coatings can function in extreme environments. We also observed that the oil separation efficiency through these membranes was as high as 99.69% and oil flux as high as 26.7 ± 5 kLm^–2^·h^–1^. A low-cost and scalable vapor-based fabrication process used to grow the nanowires on fabric demonstrates the potential for this method to be suitable for industrial processes. The ability to grow robust bioinspired FF nanowires on fabrics using a facile technique for developing hydrophobicity presents an biobased alternative material that reduces environmental footprint compared to various organic and inorganic materials currently in use.

## Supporting Information Available

Optical images of water drop on the Si substrate with and without vertical FF nanowires (Figure S1); dynamics of oil and water droplets being dropped on Si substrates with and without vertical FF nanowires (Figure S2); contact angle over time for water and oil droplets on Si substrate (Figure S3); higher-magnification (Figure S4) and lower-magnification (Figure S5) SEM images of FF nanowires on a fabric; dynamics of oil drop on the fabric with vertical FF growth (Figure S6); FF nanowire grown fabric boat for oil collection (Figure S7); and oil collected in fabric boat for over 5 days (Figure S8) (PDF)
Demonstrating oleophilicity of FF nanowire coated fabric (Movie S1) (AVI)
Demonstrating oil retrieval from fabric-based FF nanowire coated oil-water separating boat (Movie S2) (MP4)

## Supplementary Material

em4c00381_si_001.pdf

em4c00381_si_002.avi
