# Supplementary material for: Vertically Grown Bioinspired Diphenylalanine Nanowire-Coated Fabric for Oil–Water Separation
Source: ACS Appl Eng Mater. 2024 Aug 5;2(8):2219–26. doi: 10.1021/acsaenm.4c00381 (PMC11348427; doi:10.1021/acsaenm.4c00381)
Supplement: Supplementary file 1 — em4c00381_si_001.pdf [file em4c00381_si_001.pdf]

Supporting Information

Vertically Grown Bio-inspired Diphenylalanine Nanowire Coated Fabric for Oil-Water Separation

Noah Han-Deschaine[1], Neha M. Viradia[1], Jeiko J. Pujols[1,2], Sarah Miller[1], Ramesh Y. Adhikari[1,]*

[1]Department of Physics & Astronomy, Colgate University, 13 Oak Drive, Hamilton, NY 13346, USA.

[2]Department of Electrical & Systems Engineering, 200 South 33rd Street, Philadelphia, PA 19104, USA

* Email: radhikari@colgate.edu

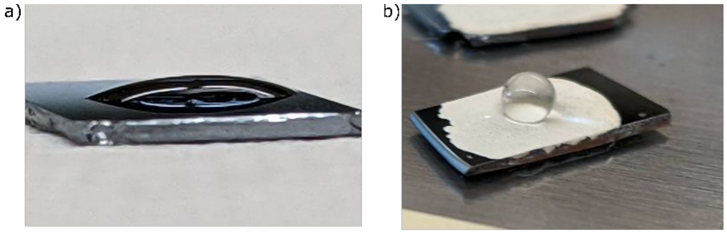

Figure S1: Optical images of water droplet on a) Si substrate with no diphenylalanine nanowire layer, b) Si substrate with vertically grown diphenylalalnine nanowire.

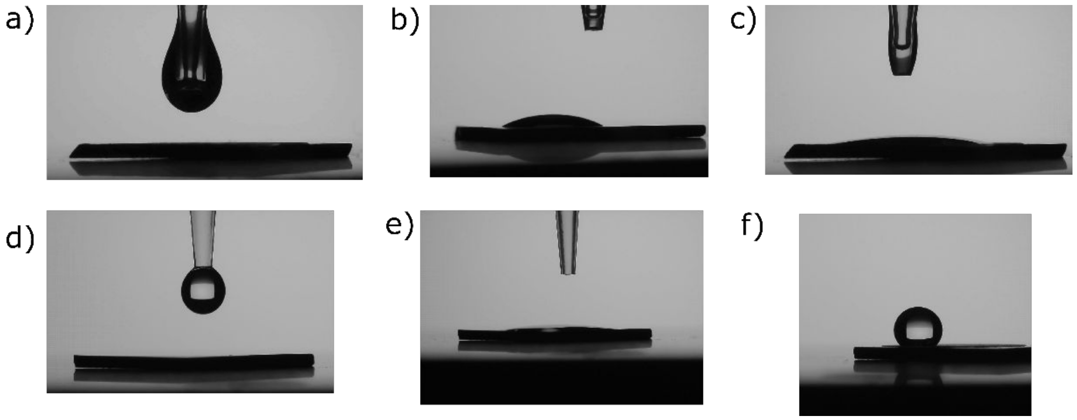

Figure S2: a) Oil Drop. b) Oil on clean Si substrate. c) Oil on FF grown Si substrate. d) Water Drop. e) Water drop on clean Si substrate. f) Water drop on FF treated Si substrate.

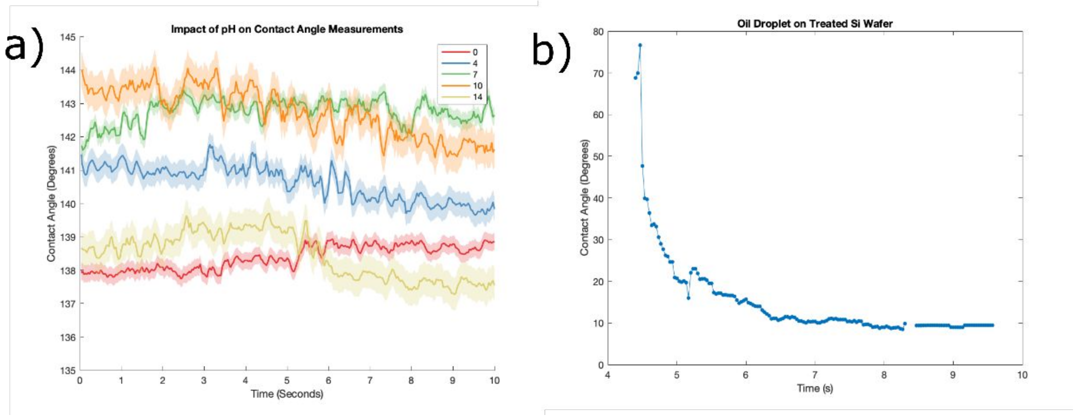

Figure S3: Contact angle over time for a) water droplet at various pHs and b) oil droplet on the diphenylalanine nanowire film grown on Si substrate.

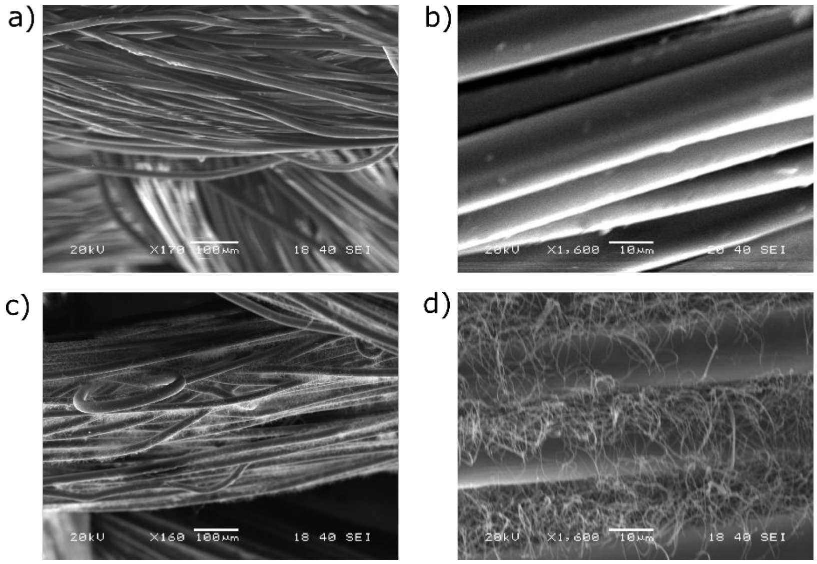

Figure S4: Surfaces of pristine fabric threads (a,b) and FF grown fabric threads (c,d) at various magnifications. (Scale bar for a,c: 100 um. Scale bar for b,d: 10 um)

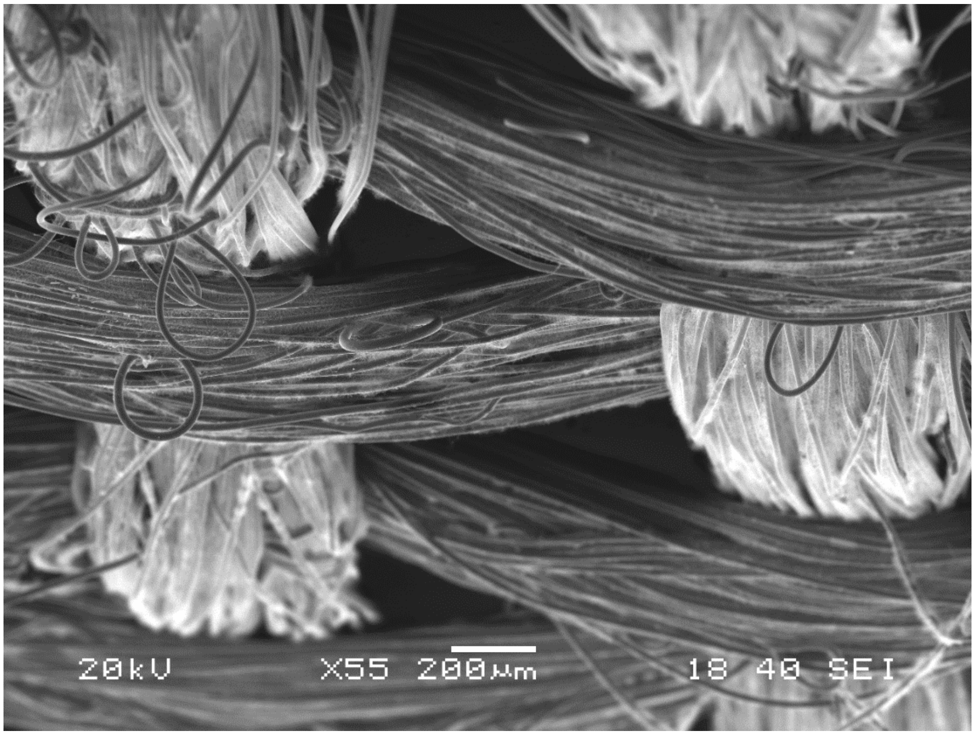

Figure S5: SEM image of the fabric with nanowire growth.

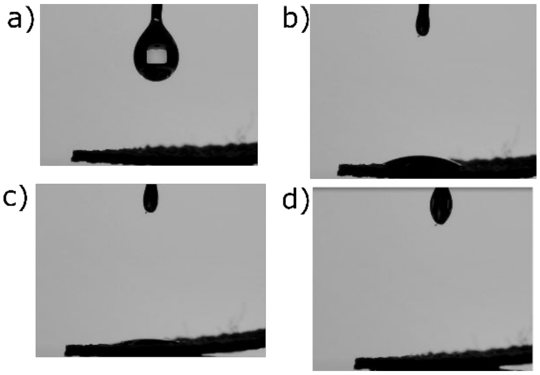

Figure S6: Demonstration of oleophilicity of fabric coated with nanowires. a) Oil drop falling from a needle. b) Oil falls on the fabric surface making a small contact angle. c) Diminishing oil drop bulge as it spreads on the surface. d) Oil drop completely dissipates from the surface. It takes only fraction of a second for these events to occur (See Movie S1).

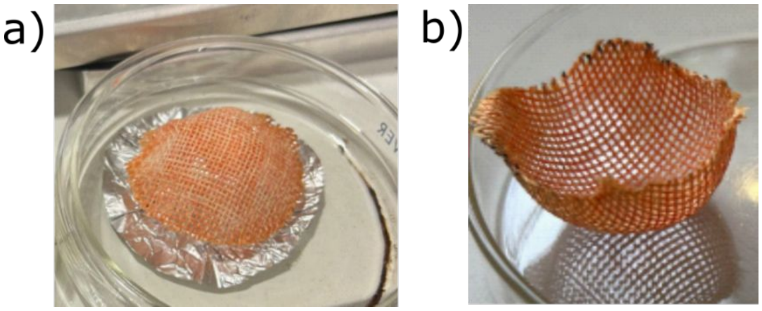

Figure S7: a) Using aluminum foil was mold for developing fabric boat before starting FF nanowire growth process. b) Fabric boat after FF nanowire growth is completed.

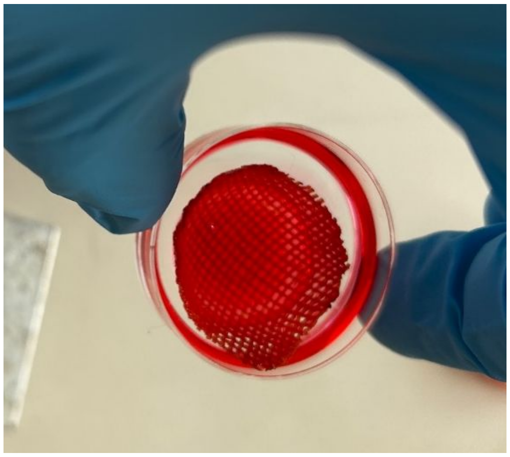

Figure S8: Oil collected with the fabric boat could be effectively retained in the boat over 5 days maintain oil-water sepration while the boat is left floating in the water.
